# Extraction and Separation of Natural Products from Microalgae and Other Natural Sources Using Liquefied Dimethyl Ether, a Green Solvent: A Review

**DOI:** 10.3390/foods13020352

**Published:** 2024-01-22

**Authors:** Tao Wang, Li Zhu, Li Mei, Hideki Kanda

**Affiliations:** Department of Chemical Systems Engineering, Nagoya University, Furocho, Chikusa, Nagoya 464-8603, Japan

**Keywords:** microalgae, green solvent, dimethyl ether, extraction, natural products

## Abstract

Microalgae are a sustainable source for the production of biofuels and bioactive compounds. This review discusses significant research on innovative extraction techniques using dimethyl ether (DME) as a green subcritical fluid. DME, which is characterized by its low boiling point and safety as an organic solvent, exhibits remarkable properties that enable high extraction rates of various active compounds, including lipids and bioactive compounds, from high-water-content microalgae without the need for drying. In this review, the superiority of liquefied DME extraction technology for microalgae over conventional methods is discussed in detail. In addition, we elucidate the extraction mechanism of this technology and address its safety for human health and the environment. This review also covers aspects related to extraction equipment, various applications of different extraction processes, and the estimation and trend analysis of the Hansen solubility parameters. In addition, we anticipate a promising trajectory for the expansion of this technology for the extraction of various resources.

## 1. Introduction

Plants contain a wide variety of naturally occurring organic compounds that are produced and metabolized in their bodies. These compounds include waxes, terpenoids, lipids, phenolic compounds, polar glucosides, alkaloids, sugars, peptides, and various other substances [1]. The most important physiologically active plant compounds include phenolic compounds (including flavonoids), saponins, and cyclins, which play key roles as dietary supplements [2]. Natural phytonutrients are widely distributed and contain a diverse range of compounds with low to high molecular weights [3,4,5]. Compounds with strong physiological activities against living organisms have attracted considerable attention from researchers, leading to the exploration of new natural products and structural modifications, particularly in fields such as medicine, pharmaceuticals, and nutraceuticals [3,4,5,6,7,8].

Organic compounds obtained from nature serve as dietary supplements that help improve health, delay aging, prevent chronic disease, prolong life, and support the structure and function of the body [9,10,11]. The market for dietary supplements was estimated to be approximately USD 353 billion in 2019 and it is growing steadily [12]. Because of the impact of the COVID-19 pandemic, the demand for dietary supplements has increased and was projected to reach approximately USD 561 billion by 2022 [13]. Consumers seek health and immune benefits from dietary supplements to protect themselves from infections and diseases.

Substances such as antibiotics, chemical preservatives, and alkaloids have been used in the formulation and extraction of bioactive compounds in various food industries, including sugarcane [14], tea [15], coffee [16], and plant extracts [17,18]. The preparation of natural material samples involves several critical steps: The initial phase includes preliminary washing of plant materials, drying or lyophilization, and grinding for homogenization. The next steps include extraction and qualitative/quantitative analyses [19]. The production of natural materials is expensive and has the drawback of reduced nutrient concentrations in the raw material itself; this poses a significant obstacle for the natural materials industry [20,21]. Consequently, several natural ingredients require solvent-based extraction and purification to produce dietary supplements [22,23].

To extract compounds from natural sources while avoiding high temperatures and dryness, researchers should employ methods that use water-miscible solvents, low-boiling solvents for extraction, or solvents that can be evaporated at 40 °C using solar heat. In addition, the selected solvents must have a large difference in boiling point compared to water to ensure minimal residue and nontoxicity. Liquefied dimethyl ether (DME) has been proposed as a solvent that meets these requirements [24].

This review provides information on the extraction of natural products from renewable sources using liquefied DME, an environmentally friendly solvent. DME, with a standard boiling point of −24.8 °C because of its molecular structure, requires pressurization up to 0.59 MPa before it can be used as a liquid solvent at 25 °C [25]. The EU allows the use of DME as a food extraction medium [26], and the United States Food and Drug Administration (FDA) classifies DME as “Generally Recognized as Safe (GRAS)” [27]. This review outlines the potential applications of liquefied DME in the extraction of various functional compounds from foods, dietary supplements, and medicinal plants. It aims to highlight how liquefied DME, as an alternative to traditional, toxic organic solvents, can be beneficial to industries engaged in the solvent extraction of natural products.

## 2. Disadvantages of Conventional Extraction Techniques

### 2.1. Disadvantages of Traditional Solvent Extraction

Conventional extraction methods for natural organic compounds have historically relied on various solid–liquid extraction techniques using organic solvents, such as the Soxhlet extraction method, immersion method, and steam distillation [28]. Commonly used solvents in these methods include acetone, acetic acid, chloroform, dichloromethane, diethyl ether, ethanol, and hexane (Table 1). The quantitative and qualitative performance of the extraction depends heavily on the polarity of the solvent used. This necessitates the selection of a solvent appropriate for the polarity of the target component, without a predefined method or solvent [29]. Despite their simplicity, conventional extraction methods suffer from low selectivity, low recovery and extraction rates, labor intensiveness, time consumption, and the use of large amounts of often toxic organic solvents, leading to potential trace residues in the extracts [30].

**Figure 1:**
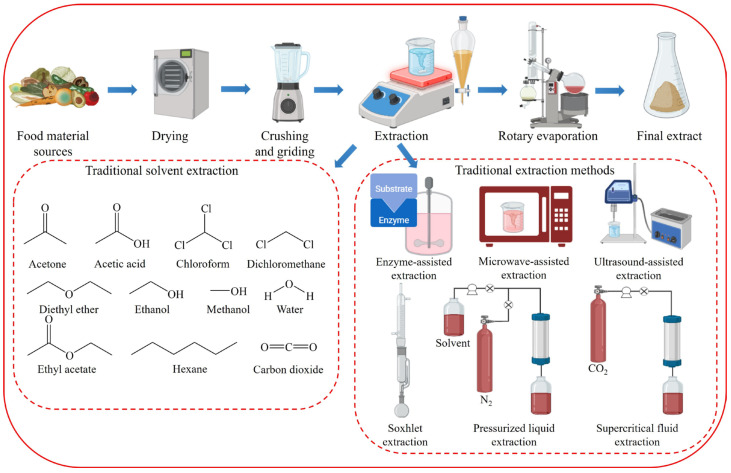
Conventional organic solvent extraction of phytochemicals.

Traditionally, organic solvents such as chloroform, hexane, methanol, and dichloromethane have been used to extract lipids and other functional compounds [31,32] (Figure 1). However, owing to significant waste generation and risks to the environment and human health, the demand for sustainable, nontoxic extraction methods has increased. Conventional extraction processes require dried algal starting materials and consume considerable time and energy [33]. For lipid extraction from microalgae, 90% of process energy consumption is attributed to lipid extraction [34]. Wet extraction, which accounts for 70% of total energy consumption, appears to be promising. Therefore, exploring new processes for lipid recovery from microalgae using limited drying methods is necessary. Currently, easily recoverable lipid species are prioritized.

Ethanol is a low molecular weight organic compound that exhibits both hydrophilic and lipophilic properties, making it suitable for the extraction of bioactive compounds such as phenolic acids, flavonoids, and phenolic acid esters [35]. However, the use of ethanol in food processing is prohibited in certain regions and cultures, necessitating the use of alternative solvents. When ethanol is used to extract highly aqueous samples, the addition of benzene to the ethanol–water mixture is required to increase water volatility and prevent water contamination via co-boiling, thereby preserving the non-polar components [36]. Considering the effect of ethanol on human health, dietary supplements should either avoid its use or maintain its concentration as low as possible [37,38]. In a study conducted in 2021, liquid herbal dietary supplements (103) were purchased from a pharmacy (Novi Sad, Serbia) and the presence of ethanol and specific residual solvents was analyzed using gas chromatography–mass spectrometry (GC–MS). Among the eighteen products for infants under two years of age, only one product had no quantifiable ethanol content [39]. Therefore, awareness among professionals and the general public should be increased.

Supercritical carbon dioxide (scCO_2_) exhibits an intermediate state between gas and liquid when maintained at high temperatures and pressures above the critical point (31.3 °C, 7.38 MPa) [40]. The use of scCO_2_ remarkably improves the extraction efficiency of functional components, enabling selective extraction via temperature or pressure control [41,42,43]. The extraction of functional components using scCO_2_ has been applied to various natural food sources, such as essential oils [44], γ-oryzanol [45], chamomile seed oil [46], and hops [47], as well as for caffeine removal [48] and oil extraction from microalgae [49,50,51]. However, the extraction of functional components from highly aqueous samples using supercritical methods is challenging [52]. This is because of the non-polar nature of scCO_2_, which often requires the addition of entrainer solvents such as methanol [53,54], ethanol [55], and acetone [56] to facilitate extraction [57].

**Table 1:** Comparison of extraction techniques using different solvents.

Solvent	Extraction Method	Extraction, Temperature, and Time	Boiling Point of Solvent [°C]	Compound Type	Reference
Acetone	Ultrasound-assisted extraction	45 °C, 20 min	56.0	Tannins	[58]
Acetic acid	Ultrasound-assisted extraction	50 °C, 30 min	118	Flavonoids	[59]
Chloroform	Homogenization and drying at 65 °C overnight	25 °C,	61.0	Lipids	[60]
Dichloromethane	Dichloromethane/methanol solvent system; freeze-drying at 80 °C for 24 h	50 °C, 6 h	39.6	Lipids	[61]
Diethyl ether	Soxhlet extraction; rinsing bath at room temperature for 12 h	5 h	34.0	Lipids	[62]
DME	Flow extraction	25 °C,	−24.8	Lipids	[63]
Ethanol	Soxhlet extraction	100 °C, 8 h	78.4	Rice bran oil	[64]
Ethyl acetate	Soxhlet extraction	8 h	77.1	Phenolic compounds	[65]
Hexane	Soxhlet extraction	60 °C, 11 h	68.7	Phenolic compounds	[66]
Methanol	Homogenization	60 °C, 24 h	64.7	Phenolics, alkaloids, flavonoids, and terpenoids	[67]
Water	Shaking incubation	25 °C, 24 h	100	Phenolic compounds, flavonoids, anthocyanins, and antioxidants	[68]

### 2.2. Disadvantages of Traditional Extraction Methods

Pre-drying is essential for moisture-containing natural products because moisture content inhibits solvent extraction [69]. For example, coffee hulls have a high moisture content, ranging from 18% to 80% *w*/*w*, which includes bound water trapped in the fine structure of solid particles. Therefore, most solvent extraction techniques require dried samples [70]. Historically, drying natural materials under natural conditions has been the primary method of preservation. However, this method is now being abandoned owing to its lengthy process and the inability to adjust the drying parameters. Recent drying methods for natural materials include heating, freezing, convection, and microwave vacuum drying [71]. Drying conditions and plant species considerably affect the content of bioactive compounds. For example, heat-drying Asiatic pennywort powder results in lower levels of phenolic bioactive compounds and reduced antioxidant activity [72]. Similarly, the vitamin C content of dried *Stevia rebaudiana* leaves decreases with increasing drying temperatures [73].

The production of instant coffee requires high thermal energy (21.10 and 8.50 MJ/kg product) for spray drying and extraction, accounting for three-quarters of the total process [74]. Spray drying is the most commonly used method for this purpose and requires 10–20 times more energy per kilogram of evaporated water than drying using an evaporator. To reduce energy consumption, researchers use evaporators to preconcentrate coffee samples before drying [75]. Heat-sensitive substances require extraction at room temperature or cold solvent removal, such as via lyophilization. High extraction temperatures result in solvent loss and component degradation. Anthocyanins, which are valuable, colored bioactive compounds, are increasingly extracted worldwide, but their functionality is limited by their decomposition at 50–60 °C, indicating limited temperature stability [76]. Essential oils, composed mainly of terpenoids and aromatic compounds, exhibit remarkable antioxidant activity but low thermal stability. Because of the low thermal stability of leaf oils, the manufacturing process of leaf-oil-related products requires the addition of antioxidants to maintain their quality [77].

The extraction of natural compounds requires a series of complex operations, such as sample drying, pulverization, extraction, and solvent removal [78] (Figure 1). The extraction process begins with solvent selection and involves the use of extraction techniques with higher extraction rates [79]. Traditional extraction techniques such as maceration, Soxhlet extraction, and decoction have significant drawbacks, including long extraction times, poor selectivity, expensive solvents, and the need for significant solvent evaporation [80]. Consequently, modern methods such as enzyme-assisted extraction (EAE), ultrasound-assisted extraction (UAE), microwave-assisted extraction (MAE), pressurized liquid extraction (PLE), and supercritical fluid extraction (SFE) have been developed [81,82]. These techniques use minimal amounts of solvents or specialized green solvents and offer several advantages over traditional extraction methods.

Maceration involves coarsely grinding the raw material, placing it in a container, pouring the solvent to completely cover the material, and extracting it while stirring until the soluble substances dissolve [83]. However, this method typically takes several days to weeks due to its long soaking time [84].

Soxhlet extraction, a model extraction technique traditionally used to extract compounds, particularly lipids, from solid or semi-solid matrices, has several drawbacks, such as long extraction times (12–24 h), high solvent volumes, high energy consumption, and issues regarding selectivity and efficiency [85,86,87].

Decoction is used to extract thermally stable bioactive compounds by boiling the raw materials in water [88]. However, this limits the extraction of water-soluble components, increases the solvent-to-solid ratio, and introduces numerous water-soluble impurities into the extract [89]. In addition, decoction cannot be used to extract thermolabile or volatile compounds.

In recent years, advances in extraction techniques have focused on modern methods that use minimal amounts of solvents or specific green solvents. EAE has proven useful for the extraction of cell-wall-associated phytochemicals [90]. This environmentally friendly method requires less energy and equipment than other techniques and reduces the use of toxic solvents. However, enzymes are prohibitively too expensive for use in large-scale extractions [91].

UAE increases the extraction efficiency by promoting solute dissolution, diffusion, and heat transfer through the generation of cavitation via ultrasound irradiation in the solvent [92]. UAE requires minimal solvent and energy consumption and reduces the extraction temperature and time. Increasing the temperature increases solute detachment from the solvent, solute solubility, solvent viscosity, and solvent diffusion within the tissue matrix. However, excessively high temperatures can decrease yield because of weakened cavitation effects [93], as observed by Al-Dhabi et al.; in this study, the yield increased with temperature from 30 to 45 °C during the extraction of phenolic compounds from used coffee grounds but decreased beyond 45 °C [94].

MAE combines traditional solvent extraction with microwave irradiation and converts the absorbed energy into heat, resulting in the heating of the material [95]. It offers advantages such as improved extraction yields, reduced thermal degradation, and the selective heating of plant materials. However, excessive microwave power can cause the decomposition of heat-sensitive compounds in the plant matrix, thereby reducing extraction efficiency [96]. In addition, MAE generates waste and consumes solvents.

PLE maintains the solvent in a liquid state above its boiling point under high pressure and increases solute solubility, diffusion, and solvent permeability into the matrix; however, its high extraction temperature (140–170 °C) may limit its use for thermolabile compounds [97].

SFE, which operates at low critical temperatures (31 °C), offers attractive advantages such as low toxicity, selectivity, inactivity, low cost, and the ability to extract thermally unstable compounds using scCO_2_, which is ideal for extracting non-polar natural products. The addition of entrainer solvents to enhance the solvation properties of CO_2_ is also feasible [98,99].

## 3. Advantages of Liquefied DME as an Extractant

### 3.1. Physical Properties of DME

DME is a simple ether with the chemical formula CH_3_–O–CH_3_ and lacks a direct C–C bond. DME has a high oxygen content (34.8%) and low carbon-to-hydrogen ratio (C:H) [100]. The two methyl groups in DME form two polarized bonds oriented at an angle of 111.8 ± 0.2°, resulting in a bent V-shaped molecular geometry around the central oxygen atom [101]. DME contains two types of bonds (C–O and C–H). Although there is a 0.4-unit electronegativity difference between C–H bonds, which results in weak polarity, C–O bonds have a 1-unit electronegativity difference, indicating a higher polarity [102]. Because of the uneven distribution of charged electron clouds throughout the molecule, DME exhibits a dipole moment of 1.3 D, making it a polar substance [103]. In addition, the polarization of the nonbonding electron pairs on oxygen contributes to the dipole moment of DME.

DME is a gas under standard conditions and has a boiling point of −24.8 °C [25]. This gaseous state results in minimal residue in the extracted materials [104]. Although denser than dry air, DME exists as a vapor at 0.1 MPa and 25 °C. Moreover, it transitions from the vapor to the liquid phase above a saturated vapor pressure of 0.59 MPa at 25 °C [105,106]. The density of liquid DME at 25 °C is 668 kg/m^3^ [107].

The dielectric constant (ε) of liquid DME at 30.5 °C and 6.3 MPa is 5.34 [108]. In comparison, the dielectric constant (ε) of water (30 °C, 25 MPa) is 80 [109]. This suggests that the polarity of DME is suitable for dissolving non-polar to moderately polar substances [110]. DME can bind to both polar and non-polar compounds via the oxygen atom at its molecular center. It forms hydrogen bonds with the hydrogen atoms of other molecules, thereby increasing extractability [63,111].

### 3.2. Cell Destruction and Drying-Free Extraction Techniques

**Figure 2:**
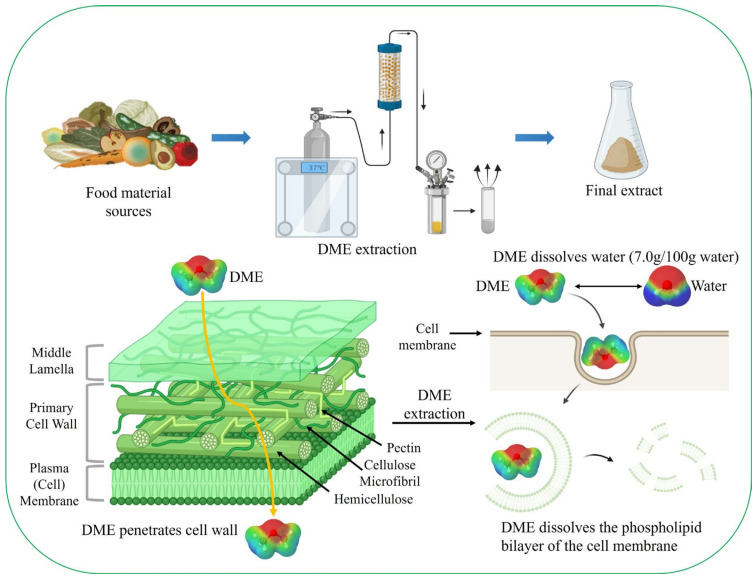
Extraction process using liquefied DME; liquefied DME dissolves the phospholipid bilayer of the cell membrane and water.

Plant cells are surrounded by a cell wall, which is mainly composed of cellulose. For example, microalgae such as *Chlorella* species possess a robust cell wall approximately 88 nm in thickness [112]. Consequently, the extraction of active components, such as lipids, from cells requires long processing times, hydrophobic extraction solvents, and energy-intensive mechanical disruption methods [113]. Because phospholipids in cell membranes are amphiphilic molecules, they require a mixture of polar and non-polar organic solvents for extraction. Liquid DME is well-known for extracting neutral and complex lipids from dairy products [114]. Liquid DME is used to extract compounds from various wet and dry biomasses containing lipid-rich compounds without cell disruption [24,115,116,117,118] (Figure 2). The liquefied DME extraction method is suitable for extracting lipids from the microalgae diatom *Chaetoceros gracilis* and the coccolithophore *Pleurochrysis carterae*, whose cell walls are biomineralized [24]. The “hard” biomineralized cell walls of the microalgae were found to have no effect on lipid extraction from liquefied DME.

In addition, an energy-intensive pre-treatment process to dehydrate the wet microalgae prior to solvent extraction is essential to increase the extraction efficiency of the active compounds in the microalgae [119]. Unlike conventional supercritical extraction solvents, DME exhibits substantial miscibility and mutual solubility in water. It exhibits high hydrophilicity and dissolves in water at 35 wt% (0.55 MPa, 25 °C), while the solubility of water in DME is 7.0 wt% (0.55 MPa, 25 °C) [120]. Pre-blending ethanol with DME at approximately 6 wt% allows for customizable mixing ratios of DME and water [120]. Upon interaction with water, DME acts as a hydrogen bond acceptor, resulting in the formation of weak hydrogen bonds between the oxygen atoms of DME and those of water [63,111]. This property allows DME to process wet feedstock [121]. Depending on the composition of the mixture, water can act as a co-solvent for water-soluble compounds [24,114,122].

### 3.3. Safety of Liquefied DME as an Extraction Solvent

DME, a recognized organic solvent with a low boiling point that is safe for human use, has significant potential for the extraction of various active ingredients. It has been approved for use as an extraction solvent in the food industries of the United States, Europe, and other regions. The European Food Safety Authority (EFSA), in its assessment of the safety of DME as an extraction solvent for the removal of fats from animal protein sources, stated that residual levels of up to 9 μg/kg in extracted animal protein do not pose a significant safety concern [26]. Food Standards Australia New Zealand (FSANZ) approved the use of DME as an extraction solvent processing aid for all dairy ingredients and products [123].

In the United States, DME can be commercially marketed for its intended use without the need for the FDA to promulgate food-additive regulations [27]. In addition, under the Federal Food, Drug, and Cosmetic Act (FFDCA), amended by the Food Quality Protection Act (FQPA) in 1996, DuPont petitioned the Environmental Protection Agency (EPA) for tolerance exemptions for DME residues. Consequently, the EPA determined that it was no longer necessary to establish a maximum residue limit for DME [124].

In experiments with rats exposed to DME, DME residues in the bodies of the rats were in the range of 14–19 ppm when the airborne concentration of DME reached 1000 ppm. These concentrations were equivalent to 1/61 of the airborne concentrations accumulated in the body. After inhalation for 60 min, the rats’ various organs showed DME concentrations that decreased to less than 4 ppm within 90 min [125,126]. In blood, exposure up to 10,000 ppm showed no significant effects, and a 30-week exposure to DME (2000–20,000 ppm) revealed no adverse effects [125,126].

As of 2022, the current toxicity data on DME lacks information on oral and ocular irritation, skin absorption, skin irritation, and skin sensitization. In 1925, Davidson et al. found that exposure to DME at concentrations of 50,000 and 75,000 ppm for 12 min produced mild intoxication but no significant objective symptoms [127]. Regarding occupational exposure limits, the accepted workplace air concentration for DME in Europe, the United States, and Japan is 1000 ppm, which is equivalent to the handling standards for liquefied petroleum gas (LPG) [125,126]. DuPont recommends an exposure limit (eight-hour, time-weighted average) of 1000 ppm (*v*/*v*) for DME in the workplace [128].

### 3.4. Environmental Issues Caused by Liquefied DME Extraction

The synthesis of DME from renewable sources, utilizing biomass-derived CO_2_ and hydrogen generated via water electrolysis (powered by solar or wind energy), enables the production of DME from renewable feedstocks [129]. The lack of explosive peroxide formation in DME allows safe storage [130]. As it does not form peroxide aerosols, DME has attracted considerable attention as a propellant for household hairsprays [131]. Generally considered biodegradable, nontoxic, non-carcinogenic, and non-corrosive, DME has proven to be ideal for various everyday applications such as personal care products (hairsprays, shaving creams, foams, and antiperspirants), household products, paints, coatings, food, insect repellents, and animal products [132,133]. DME then undergoes photochemical reactions with OH radicals to produce CO_2_ and H_2_O [134]. Experimental modeling under ultraviolet radiation indicates that DME has a degradation half-life of 3–30 h, reaching approximately 100–150 h in the upper atmospheric regions up to an altitude of approximately 10 km. Although freon compounds may take several years or decades to degrade, DME degradation occurs in approximately 0.014 years (5.1 days) [134]. Owing to its atmospheric degradation time of several tens of hours, DME contributes minimally to photochemical reactions and is, therefore, of negligible concern regarding global warming or ozone depletion [135]. Reports indicate that DME has a global warming potential of 1.2 over 20 years, 0.3 over 100 years, and 0.1 over 500 years for CO_2_ [134]. With a short atmospheric life of 5.1 days and negligible ozone impact, DME is a spray gas that does not destroy the ozone layer [136]. DME has several advantages such as nontoxicity and low environmental risk, ensuring that it does not contaminate water, sink into waterways, or leach into the soil, even in the event of accidental release.

DME has similar physical properties to LPG and has been developed as a synthetic fuel. In China, it is used commercially as a substitute for LPG in city gas, often blended with 20% propane for consumer use [137]. It is also used as a fuel in automotive and industrial applications. However, pure DME has an explosive range of 3.427 vol% in air, which is a significant safety concern when used as an extraction solvent [137]. To address this issue, the blending of DME with CO_2_ has been investigated. When the mole fraction of CO_2_ exceeds 0.882, the mixture falls out of the explosive range and becomes non-flammable [137]. In addition to flammability concerns, DME offers unique extraction capabilities. DME, although weakly polar, has the ability to extract both polar and non-polar substances, unlike supercritical carbon dioxide and hexane that are non-polar and selectively extract non-polar substances [137].

### 3.5. Liquefied DME Extraction 

**Figure 3:**
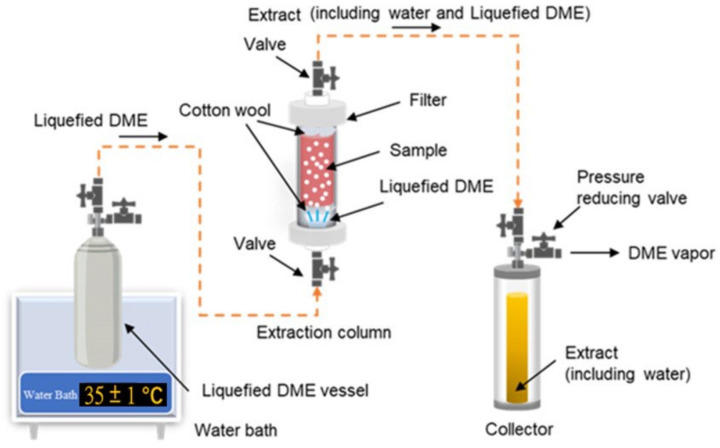
Schematic of a laboratory-scale extraction system using liquefied DME.

Figure 3 shows a schematic of a laboratory-scale liquefied DME extraction system [138]. This DME extraction system consists of a series of connections, including a metal tank containing liquefied DME (500 mL capacity), an extraction column (10–100 mL capacity), and an extraction solution collection tank (96 mL capacity).

**Figure 4:**
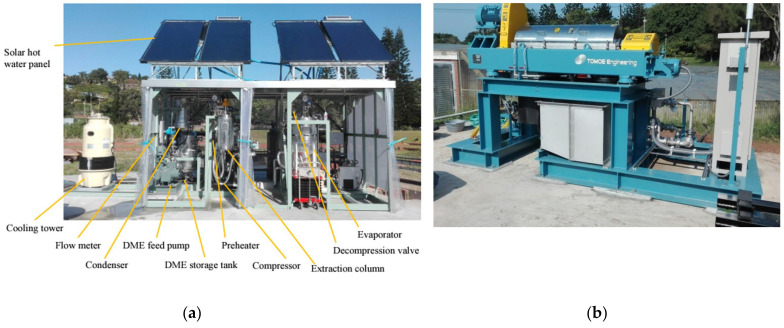
(**a**) Experimental setup of a microalgae oil extraction system using liquefied DME; (**b**) A microalgae recovery system [140].

Kanda et al. pioneered the design and development of the first DME ambient-temperature drying and purification process prototype [139]. Using this prototype, ambient temperature dewatering and deodorization of high-moisture coal and sewage sludge were achieved [139]. Kanda et al. (2019) developed the largest microalgal oil extraction apparatus in the world (Figure 4a). This apparatus successfully extracts oil from high-moisture microalgae without drying [140]. In addition, they were able to limit CO_2_ emissions during the extraction process to a level determined by the CO_2_ captured from the oil. A centrifugal separator was used to recover microalgae from a 300-ton raceway cultivation tank at 1500–2100 G and at a processing rate of 3–7 tons per hour (Figure 4b).

As shown in Table 2, modular and pilot-scale extraction systems have been developed and are sold worldwide for the extraction of useful components using liquefied DME. The Dexso Butanex 345/600 mm extractor is a modular aluminum extraction system [141]. This method can be used to directly produce high-purity essential oil extracts from plants. Other extractors are designed for solvents with a boiling point below ambient temperature, such as liquefied DME or butane, typically with a boiling point below 0 °C and a liquefaction pressure below 0.8 MPa at 20 °C [142,143,144]. These are characterized by the fact that the vaporized solvents are recompressed and liquefied for reuse. Because its body volume is as large as 2.5–200 L, the extractor can be installed in various places, such as laboratories, manufacturing plants, food and beverage factories, and agricultural food stores. The specific heat capacity of liquefied DME at 310 K and 0.817 MPa is 2.70 kJ·kg^−1^·K^−1^ [145]. The latent heat of vaporization of DME is 460 kJ·kg^−1^ [146]. In contrast, the specific heat capacity of methanol at 293.15 K is 2.55 kJ·kg^−1^·K^−1^. However, the latent heat of vaporization of methanol is 1109 kJ·kg^−1^ [147]. Because the latent heat of vaporization of methanol is much greater than that of DME, recycling the same weight of liquefied DME would theoretically reduce the energy consumption to less than half that of methanol.

## 4. Applications of Liquefied DME Extraction 

### 4.1. Lipid Extraction from Microalgae 

Microalgal biomass is a rich source of various nutrients, including fatty acids, carotenoids, proteins, minerals, and other essential nutrients that can be used as functional food ingredients [140,141,142,143,144,145,146,147,148]. Many species of microalgae grow well in saline water, such as seawater, thus avoiding the need for limited freshwater resources [149]. Some oleaginous species of microalgae overproduce lipids and fatty acids by modifying the physical and chemical properties of the culture medium [150]. The lipid content of some microalgae may reach 77%, exceeding the index of higher plants (such as soybeans) [151]. The protein content of *Arthrospira maxima* has been found to reach 71% [152].

Microalgal oils have recently been used as alternatives to fish and vegetable oils with low nutritional values [153]. Microalgal oils contain highly utilizable and nontoxic fatty acids, such as polyunsaturated fatty acids (PUFA), arachidonic acid (ARA), c-linolenic acid (GLA), eicosapentaenoic acid, and docosahexaenoic acid (DHA) [154]. Long-chain polyunsaturated fatty acids such as eicosapentaenoic acid (ω-3 C20:5) and DHA (ω-3 C22:6) obtained from microalgae are essential for humans due to their beneficial effects on health, including neurodevelopment and the prevention of chronic diseases [155]. Certain microalgal species (Haptophyta, Bacillariophyta, Ochrophyta, and Rhodophyta) have been reported to contain up to 30–50% eicosapentaenoic acid [156,157,158,159] and 22–58% DHA of the total fatty acids [160]. 

Table 3 summarizes examples of studies on lipid extraction from microalgae using liquefied DME. In 2010, Kanda et al. successfully extracted oils from high-moisture natural blue–green microalgae (91% moisture) at 20 °C using liquefied DME for the first time [161]. They extracted 40.1% of the sample dry weight lipids by flowing 114 g of liquefied DME at 10 mL/min over 6.650 g of natural blue–green microalgae (0.599 g dry sample). In addition, 68.1% of water was simultaneously extracted and 99.7% of the lipids were extracted into liquefied DME, compared to the Bligh–Dyer method of total lipid extraction. This study demonstrated the possibility of extracting lipids directly from wet microalgal cultures.

Kanda et al. observed that the extraction of liquefied DME from five microalgae species (78.2–93.4% moisture content) yielded 9.9–40.1% of the dry weight of the microalgae [162]. The extraction of lipids using the liquefied DME extraction and Bligh–Dyer methods was comparable. Analysis of the molecular weight distribution of the extracted lipids via gel permeation chromatography (GPC) showed that the liquefied DME and Bligh–Dyer methods were comparable for chloroform and tetrahydrofuran eluents. The weight-average and number-average molecular weights obtained using the liquefied DME method were similar to the molecular weight distribution of the lipids extracted using the Bligh–Dyer method. A disadvantage of the degradation method in terms of fuel quality is the high N content due to the gasification of chlorophyll and proteins at high temperatures (300–600 °C) [163]. The lipids extracted using DME had low oxygen and nitrogen contents (2.62%) and high carbon (70.9%) and hydrogen (10.0%) contents. The higher heating value (HHV) of the extract was relatively high (33.8 MJ/kg), which is comparable to the HHV of first-generation biodiesel and essentially the same as that of conventional fossil oil [162,164]. Furthermore, the HHV of the residue after DME extraction was 18.3 kJ/kg, but the residue retained sufficient heat content to be a potential carbon-neutral fuel.

The paste derived from green alga *Botryococcus braunii* Race B has attracted considerable attention as a petroleum substitute because it has a high hydrocarbon content (25–75% dry weight), and its hydrocarbon components, botryocoxene, and methylqualene can be easily converted into biofuels [165,166]. *Botryococcus braunii* also secretes extracellular hydrocarbons, a feature not observed in other algae. Liquefied DME was used to extract hydrocarbons and lipids from the *Botryococcus braunii* Race B paste [167]. The extraction yield and major components of the *Botryococcus braunii* Race B paste extract obtained via liquefied DME extraction were similar to those obtained using hexane Soxhlet extraction of the dried mass; GCMS of the DME extract showed that the major components of the *Botryococcus braunii* Race B paste, the C32–C34 botryococcenes, were present in large amounts.

The successful use of liquefied DME-based lipid extraction has been reported for several microalgae species, including *Haematococcus pluvialis* [168], *Euglena gracilis* [169,170], *Aurantiochytrium limacinum* [171], *Arthrospira platensis* [172], *Chaetoceros gracilis, Pleurochrysis carterae* [24], *Monostroma nitidum* [173], *Tetradesmus obliquus* [173], *Nannochloropsis oculate* [174], *Phaeodactylum tricornutum* [175], *Haematococcus pluvialis* [176], and various others [177,178]. These extractions resulted in high yields of specific oleaginous components and bio-oils, highlighting the efficacy of liquefied DME extraction.

Recent studies on common microalgae and cyanobacterial species (*Arthrospira platensis, Nannochloropsis gaditana, Phaeodactylum tricornutum,* and *Scenedesmus almeriensis*) using dried powders for liquefied DME extraction have reported oil yields ranging from 0.5% to 2.7% of the dried mass (5–19% of total lipids) [177]. At the same time, cryo-milling of algae increased lipid yields to 1.7–5.6% of the dry mass (17–50% of total lipids), including valuable polyunsaturated fatty acids influenced by the microalgae species. The ease of lipid separation and high dehydration capacities of liquefied DME-based lipid extraction make it a promising method for lipid extraction from microalgae.

**Table 3:** Extraction of lipids from microalgae using liquefied DME: lipid and water extraction rates.

Authors	Resource	Lipid Extraction Yield (%)	Water Content (%)	Dewatering Rate (%)
Kanda et al., 2011 [161]	Natural blue–green microalgae	40.1	91.0	68.1
Kanda et al., 2012 [162]	Natural blue–green microalgae	9.9–23.2	78.2–93.4	83–91
Kanda et al., 2013 [167]	*Botryococcus braunii* Race B paste	48.9	74.3	–
Boonnoun et al., 2014 [168]	*Haematococcus pluvialis*	30.0	82.1	–
Kanda et al., 2015 [169]	*Euglena gracilis*	32.5	80.3	92.0
Sakuragi et al., 2016 [170]	*Euglena gracilis*	19.7	95.0	–
Hoshino et al., 2016 [171]	*Aurantiochytrium limacinum*	46.1	67.9	–
Hoshino et al., 2017 [172]	*Arthrospira platensis*	9.8	80.1	94.2
Kanda et al., 2020 [24]	*Chaetoceros gracilis* *Pleurochrysis carterae*	22.0 11.6	88.5 62.0	81 –
Wang et al., 2020 [173]	*Tetradesmus obliquus*	21.9–29.5	65.0–85.0	100
Wang et al., 2021 [174]	*Nannochloropsis oculata*	23.3	94.8	100
Bauer et al., 2022 [175]	*Phaeodactylum tricornutum*	9.2	10.0–80.0	–
Myint et al., 2023 [176]	*Haematococcus pluvialis*	290.1 mg g^−1^ dry extract	75.7	99.3
Bauer et al., 2023 [177]	Four common microalgae and cyanobacteria	1.7–5.6	4.70–2.51	–
Kanda et al., 2023 [178]	*Chaetoceros simplex var. calcitrans*	22.7	90	100

### 4.2. Extraction of Functional Components from Natural Resources

In addition to lipids, liquefied DME has been used to extract bioactive compounds from various sources, including spices, green tea, algae, fruits, vegetables, grains, natural plants, and fish (Table 4). In 2003, Catchpole et al. used liquefied DME to extract specific pungent compounds from ginger, black pepper, and chili powder [179]. Despite the significant extraction of water, liquefied DME showed similar efficacy as scCO_2_ in isolating pungent compounds from spices. Complete extraction was achieved with minimal solvent consumption. At temperatures of 35, 40, 50, and 60 °C, liquefied DME showed nearly equivalent extraction rates. Subsequently, subcritical propane was suggested as a cost-effective alternative to CO_2_ because of its lower operating pressure and reduced energy consumption during spice extraction, similar to liquefied DME. However, subcritical propane is the least effective at dissolving pungent components and is unsuitable for carotenoid extraction [179,180].

Liquefied DME has been previously used to decaffeinate green tea [181]. The main functional components of green tea are caffeine and catechins [182]. Excessive caffeine consumption can lead to health problems, such as dizziness, increased heart rate, tremors, and insomnia, owing to overstimulation of the central nervous system [183]. Liquefied DME enables catechin extraction while completely removing caffeine. Ciulla et al. also demonstrated higher extraction rates of caffeine from coffee beans and powder using liquefied DME rather than using scCO_2_-based extraction methods [184].

Natural carotenoids exhibit several beneficial effects, including antioxidant, anti-inflammatory, antiproliferative, and antiapoptotic properties [185]. As antioxidants, carotenoids detoxify intracellular free radicals, thereby reducing the incidence of oxidative damage and associated diseases [186]. Carotenoids, which are widely distributed in nature, are biosynthesized by various organisms, including photosynthetic organisms such as algae, plants, fungi, and bacteria [186]. As humans lack the ability to synthesize carotenoids internally, their intake of carotenoids is primarily through fruits and vegetables, plants, and algae.

Using an enzyme-assisted DME and ethanol co-solvent extraction method, Billakanti et al. were able to extract almost all lipids, including polyunsaturated fatty acids and fucoxanthin, from the wet, brown seaweed *Undaria pinnatifida* [187]. *Undaria pinnatifida* contains a mixture of sulfated and branched chain polysaccharides that are tightly bound to the cell wall [188]. Therefore, extracting bioactive compounds from brown seaweed biomass is difficult because the cell wall is a major obstacle [189]. Kanda et al. successfully extracted high concentrations of fucoxanthin (390 µg/g dry *Undaria pinnatifida*) from wet *Undaria pinnatifida* (water content was 93.2%) using liquefied DME (286 g, extraction time 43 min) [111]. This yield was significantly higher than those achieved using Soxhlet extraction with ethanol (50 µg/g) and scCO_2_ extraction (60.12 µg/g) [190].

Microalgae have attracted widespread attention as natural sources of carotenoids because they grow faster than other higher plants. The Liquefied DME extraction method successfully extracted 7.70 mg/g of astaxanthin, a carotenoid, and 30.0% of its dry weight of lipids from microalgae (*Haematococcus pluvialis*) [168]. The extraction rate of astaxanthin was 1.82% lower than that achieved through acetone extraction using drying and cell disruption. Liquefied DME extraction removed 92% of the water from the microalgae and increased the carbon and hydrogen contents. Babadi et al. reported the extraction of total carotenoids (4.14 mg/g algal dry weight) and total chlorophyll (8.45 mg/g algal dry weight) from the microalgae *Chlorococcum humicola* using liquefied DME [191]. Liquefied DME extraction was performed using a liquefied DME: algae wet weight ratio of 45:1 (*w*/*w*) at 41 °C for 20 min with stirring at 400 rpm. Liquefied DME showed a higher extraction rate than the conventional solvent acetone, suggesting that it is highly selective toward less polar carotenoids. Liquefied DME has also been successfully used to extract carotenoids from other raw materials, such as Japanese pumpkin peel [192] and marigold flowers [193].

Rice bran has been reported to have cholesterol-lowering and antioxidant properties [194]. Γ-Oryzanol, a bioactive compound abundant in rice bran, has been reported to have antioxidant, anti-inflammatory, anticancer, and antidiabetic effects [195]. Liquefied DME has been reported to extract γ-oryzanol; unsaturated fatty acids such as linoleic and oleic acid, and phytosterol; and the plant wax extract policosanol from rice bran [45,117,196].

**Figure 5:**
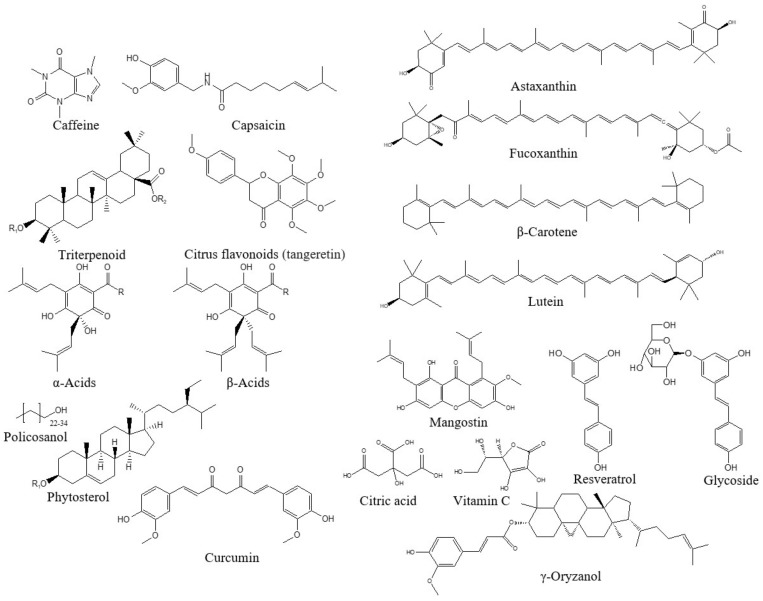
Structure of bioactive compounds extracted from natural products using liquefied DME.

Moreover, liquefied DME has been used to extract bioactive compounds from citrus leaves and peels (citrus flavonoids) [197], *Garcinia mangostana* Linn (mangostin) [198], vegetables (proteins) [199], lemon peel tissue (citric acid, vitamin C, and essential oils) [200], tuna liver (fish oil, n-3 polyunsaturated fatty acids) [201,202], macroalgae *Monostroma nitidum* (Lutein) [203], Japanese knotweed rhizomes (resveratrol and glycoside) [204], *Centella asiatica* leaves (triterpenoid) [205], hops (α-acids and β-acids) [206], cyanobacteria (fatty acids) [207], sugar mill waste (policosanol and phytosterol) [208], *Curcuma longa* L. (curcumin) [138], and diatom *Chaetoceros simplex var. calcitrans* (fucoxanthin) [178] (Figure 5). Most studies have indicated that liquefied DME exhibits a higher extraction rate than conventional extraction methods. In addition, liquefied DME, which is nontoxic and leaves no residue in the extract, is one of the best extraction solvents because it extracts bioactive compounds directly from wet natural products without the need for drying, grinding, or other manipulations.

**Table 4:** Extraction of biologically active compounds from natural products using liquefied DME extraction.

Authors	Resource	Specific Ingredients	Extraction Solvent	Lipid Extraction Yield (Dry Weight of the Microalgae)
Catchpole et al., 2003 [179]	Chili, black pepper, and ginger	Capsaicin	Liquefied DME scCO_2_ Propane Acetone	19 g/kg 19 g/kg 11 g/kg 20 g/kg
Kanda et al., 2013 [181]	Green tea	Caffeine	Liquefied DME	47 μg/g
Billakanti et al., 2013 [187]	Macroalgae (*Undaria pinnatifida*)	Fucoxanthin	Liquefied DME Ethanol	0.066 mg/g 0.060 mg/g
Hoshino et al., 2014 [197]	Citrus Leaves and Peels	Citrus flavonoids	Liquefied DME	6.6–49.9 mg/g
Boonnoun et al., 2014 [168]	Microalgae (*Haematococcus pluvialis)*	Astaxanthin	Liquefied DME Acetone	0.33% 1.82%
Kanda et al., 2014 [111]	Macroalgae (*Undaria pinnatifida*)	Fucoxanthin	Liquefied DME	390 μg/g
Goto et al., 2015 [190]	Macroalgae (*Undaria pinnatifida*)	Fucoxanthin	Liquefied DME scCO_2_	390 μg/g 58 μg/g
Noriyasu et al., 2015 [192]	Japanese squash peel	Chlorophylls and carotenoids	Liquefied DME	0–300 μg/g fresh weight
Nerome et al., 2016 [198]	*Garcinia Mangostana* Linn	Mangostin	Liquefied DME Ethanol	42.9 mg/g 41.14 mg/g
Boonnoun et al., 2017 [193]	Marigold flowers	Lutein	Liquefied DME	20.65 mg/g
Furukawa et al., 2016 [199]	Vegetable	Proteins	Liquefied DME	–
Nakamura et al., 2017 [200]	Lemon peel tissue	Citric acid Vitamin C Essential oils	Liquefied DME	10.75 mg/100 g 43 mg/100 g 4%
Fang et al., 2018 [201]	Tuna liver	Fish oil	Liquefied DME scCO_2_	17.46 ± 0.23% 17.51 ± 0.11%
Kerdsiri et al., 2020 [117]	Jasmine rice bran	γ-oryzanol Linoleic acid Oleic acid	Liquefied DME	2.47% 22.4% 39.5%
	Rice berry and rice bran	γ-oryzanol Linoleic acid Oleic acid	Liquefied DME	6.01% 20.0% 33.5%
Fang et al., 2019 [202]	Tuna livers	n-3 Polyunsaturated fatty acids	Liquefied DME Wet reduction Enzymatic extraction scCO_2_	98.57 ± 0.60% 56.76 ± 1.57% 85.25 ± 1.29% 98.45 ± 1.04%
		Vitamins	Liquefied DME Wet reduction Enzymatic extraction scCO_2_	37.91 μg/g 17.99 μg/g 24.43 μg/g 40.26 μg/g
Wongwaiwech et al., 2020 [196]	Rice bran oil	γ-oryzanol Phytosterol Policosanol	Liquefied DME	924.51 mg/100 g 367.54 mg/100 g 30,787 mg/100 g
Babadi et al., 2020 [191]	*Chlorococcum humicola*	Carotenoids Chlorophylls	Liquefied DME	4.14 mg/g 8.45 mg/g
Kanda et al., 2020 [203]	Macroalgae	Lutein	Liquefied DME	0.30 mg/g
			Chloroform−methanol extraction	0.24 mg/g
Kanda et al., 2022 [204]	Japanese knotweed rhizome	Resveratrol and glycoside	Liquefied DME Ethanol	0.342 and 2.57 mg/g 0.215 and 2.01 mg/g
Pingyod et al., 2021 [205]	*Centella asiatica* leaves	Triterpenoid	Liquefied DME and ethanol	18.80%
Bizaj et al., 2021 [206]	Hops	α-Acids	Liquefied DME Propane scCO_2_ Sulfur hexafluoride	9.6% 8.7% 7.9% 0.1%
		β-Acids	Liquefied DME Propane scCO_2_ Sulfur hexafluoride	4.5% 4.3% 3.8% 0.1%
Li et al., 2021 [207]	Cyanobacteria	Fatty acids	Liquefied DME	8.72–21.15%
Kamchonemenukool et al., 2022 [208]	Sugar mill waste	Policosanol	Liquefied DME	2888 mg/100 g
		Phytosterol		10,147.75–20,878.75 mg/100 g
Ciulla et al., 2023 [184]	Coffee beans and powder	Caffeine	Liquefied DME scCO_2_	0.479 mg/g 0.32 mg/g
Kamchonemenukool et al., 2023 [45]	Rice bran acid oil	γ-oryzanol	Liquefied DME scCO_2_	4865.25 mg/100 g, 2569.04 mg/100 g
Kanda et al., 2023 [138]	*Curcuma longa* L.	Curcumin	Liquefied DME	7.94 mg/g
			Ethanol	6.77 mg/g
Kanda et al., 2023 [178]	*Chaetoceros simplex var. calcitrans*	Fucoxanthin	Liquefied DME	9.2 mg/g
			Ethanol	11.9 mg/g

## 5. Theoretical Study of Liquefied DME 

The use of Hansen solubility parameters (HSP) to evaluate the solubility of various analytes of natural origin has increased [209]. HSP is used to quantify molecular interactions and solubility [210,211].

HSP is based on three interaction forces: dispersion, dipole, and hydrogen bonding forces. The dispersion force (δd) indicates random interactions between molecules and represents the non-polar nature of the molecules. The dipole force (δp) indicates polar interactions between molecules and represents the polar nature of the molecule. The hydrogen bonding force (δh) represents hydrogen bonding interactions between molecules and their hydrogen bonding abilities [212]. These interaction forces can be summed to obtain the HSP. The solubility of a substance in a solvent is higher when its HSP is similar to that of the solvent.

HSPs are typically estimated using experimental data or molecular modeling techniques [213,214]. The HSP distance between two substances is expressed by the following equation [215]:

(1) $$R_{a}=\sqrt{{4\left(∆\delta d\right)}^{2}+{\left(∆\delta p\right)}^{2}+{\left(∆\delta h\right)}^{2}}.$$

The difference in the HSP R_a_ [MPa^1/2^] can be obtained by taking the sum of the squares of the differences between the three parameters and determining their square roots [213]. The smaller the difference, the more similar the interactions between the substances and the higher the solubility and compatibility.

Based on the experimental data on solute–solvent interactions, plotting the solubility parameters of good and poor solvents for a solute in a three-dimensional diagram produces a Hansen solubility sphere, with regions of good solvents clustered together [209]. The spherical region indicates the extent to which the substance interacts with the solvent. The radius of the sphere represents the interaction radius R_0_ [MPa^1/2^]. The ratio of R_a_ to R_0_ is the relative energy difference (RED), which can be calculated using Equation (2). Here, RED ≤ 1 indicates a good solvent and RED > 1 indicates a poor solvent. RED can be used as an indicator of solubility [209].

(2) $$RED=\frac{R_{a}}{R_{0}}$$

**Figure 6:**
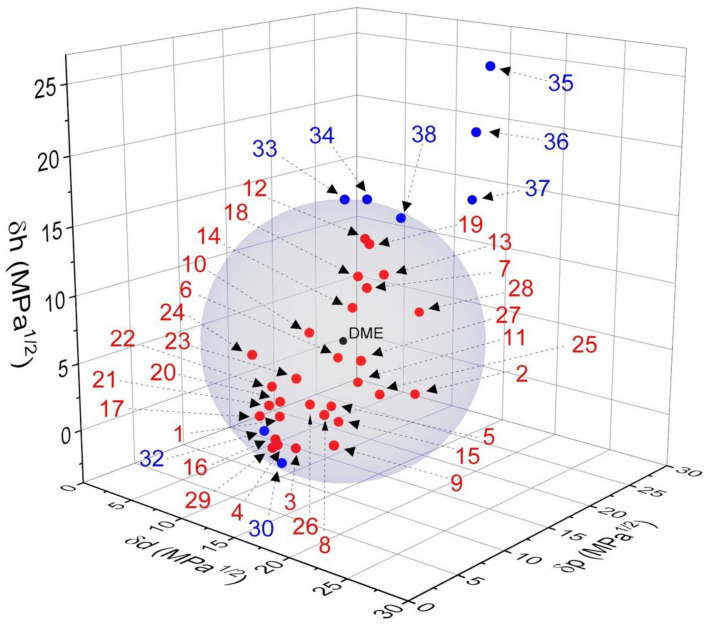
HSP sphere model for liquefied DME (Red dots indicate dissolved components inside the sphere, blue dots indicate insoluble components outside the sphere, and black dots indicate the center of the DME sphere. Numbers correspond to compounds in Table 5).

In this study, Hansen solubility spheres for liquefied DME were generated using the dissolved (29 species) and insoluble (9 species) components of liquefied DME (Table 5). The HSP values of components that reliably dissolved in liquefied DME were obtained from the literature. These extractable components are listed in Table 4. Some components were calculated using the SMILES string method based on their molecular structures [216]. The HSPs of some polymers were calculated by performing dissolution experiments. Spheres were calculated by assigning their HSP data to the HSPiP software 4.1.04 [217]. As shown in Table 5 and Figure 6, the δd, δp, and δh of liquefied DME were 19.2 MPa^1/2^, 6.3 MPa^1/2^, and 9.2 MPa^1/2^, respectively, while its R_0_ was 9.4 MPa^1/2^. Furthermore, the calculated RED values for liquefied DME and the solute are in good agreement with the actual solubility experiments. Accurate calculation of the HSP of liquefied DME is important for understanding the solubility of the target components during solvent extraction.

## 6. Bioactive Extraction to Biomedical Advances

Tuna are one of the most important marine fish species worldwide [240]. Tuna giblets are rich in bioactive compounds such as unsaturated fatty acids, vitamins, and proteins. These compounds have antioxidant properties and can be converted into value-added products [241]. However, the internal organs, particularly the livers, of tuna are difficult to process and are often discarded [242].

Fang et al. reported that liquefied DME can be used to extract lipids and vitamins from tuna liver [202]. Compared to the conventional scCO_2_ method, liquefied DME extraction can prevent lipid oxidation and effectively reduce damage to omega-3 polyunsaturated fatty acids (n-3 PUFAs) and vitamins, thereby obtaining high-quality liver oil with excellent yield. The pressure used in liquefied DME extraction is much lower (0.8 MPa) than that used in scCO_2_ extraction (35 MPa), and no freeze-drying pretreatment is required.

Lipids, water, and vitamins can be extracted from tuna liver using liquefied DME to precipitate high-quality proteins. Currently, pH shifts, including alkaline or acidic extraction, isoelectric precipitation, centrifugation, and lyophilization, are the best processing methods for obtaining proteins from tuna liver [243]. However, this method is complex and time-consuming, and the lyophilization process is time- and energy-intensive [244]. Fang et al. used liquefied DME to extract lipids, pure metals, and water from tuna liver and successfully isolated a high-quality protein powder [245,246]. The protein powder remaining in the extraction residue demonstrated the superior ability of liquefied DME extraction over conventional methods because its structure remained unaltered. However, the protein powder contained a few toxic substances. Liquefied DME extraction removes oils and fats from sturgeons and produces high-quality protein powder [247,248]. In conclusion, liquefied DME extraction has proven to be a promising low-cost technology for the fish-oil industry. This technique is capable of extracting value-added unsaturated fatty acids and vitamins and produces high-quality protein powder in the residue.

Kanda et al. crystallized glycine from an aqueous solution using liquefied DME as an antisolvent [249]. Liquefied DME can be operated at 20–25 °C, potentially reducing the energy consumption of drying or crystallization with ethanol. Kanda et al. also prepared liposomes by dissolving soy lecithin and cholesterol in liquefied DME and infusing them into warm water [250]. Transmission electron microscopy, dynamic light scattering for particle size distribution measurements, and zeta potential measurements revealed that the resulting liposomes ranged in size from approximately 60 to 300 nm, with a zeta potential of approximately −57 mV. This indicates that the liquefied DME injection method successfully produces liposomes similar to those produced using conventional diethyl ether at temperatures above 45 °C. The liquefied DME method does not require the residue of conventional diethyl ether in the final product of liposomes or the high-temperature and high-pressure conditions of scCO_2_.

Organ transplantation is a treatment option for patients with severe organ failure. During organ transplantation, cells derived from the patient are grown on a three-dimensional scaffold to create an organ that will not be rejected. When porcine tissue is decellularized to create a scaffold, the porcine aorta is similar in structure to the human aorta, making it suitable for transplantation into humans [251]. The decellularization of tissues from different species involves three steps: extraction of lipids using sodium dodecyl sulfate (SDS), DNA fragmentation using DNase, and the removal of DNA fragments via washing with water and ethanol [252]. However, long processing times, inflammation caused by SDS at the contact site, and difficulty in completely removing the toxic surfactant from the tissue may cause certain problems. Liquefied DME was used to extract lipids, DNA, and cell nuclei from ostrich carotid tissue and porcine aorta [252,253,254,255]. Demonstrating that ostrich carotid tissue can be used as an alternative to porcine scaffolds, researchers can decellularize the porcine aorta after lipid extraction using DME, followed by DNase treatment and washing for at least five days. Furthermore, the introduction of liquefied DME into conventional decellularization eliminates the need for surfactants.

## 7. Future Trends

Hypersaline brines are produced via various processes, including oil and gas production, and can contaminate surface water and groundwater if improperly treated [256]. High-pressure reverse osmosis (HPRO) is typically used to remove high salt concentrations [257]. However, salinity is proportional to the pressure required for desalination; therefore, RO with high osmotic pressure has high energy costs and requires high pressure (100–300 bar) [258].

Another method for producing hypersaline brines is distillation, which is energy intensive because it requires a phase change in water [259]. Desalination of hypersaline brines can recover valuable minerals from seawater and industrial wastewater while reducing the environmental risks associated with disposal [260].

The recent solvent-based technology for desalinating hypersaline brines offers the advantage of avoiding the high thermal evaporation of water during extraction and regeneration. Additionally, it does not face the practical limitations associated with membrane systems in comparison to conventional methods [261]. Two different processes are used in this method: solvent-driven water extraction (SDWE) and solvent-driven fractional crystallization (SDFC) [262].

In SDWE, a water-soluble organic solvent is poured into industrial wastewater or seawater to increase the concentration of inorganic salts in the aqueous solution, which are then precipitated [263]. The recovery of desalinated water does not use conventional evaporation techniques but utilizes low-energy phase transfer through a solvent–water liquid equilibrium or vapor–liquid phase equilibrium. Therefore, SDWE is used for seawater desalination [263].

SDFC, also known as antisolvent crystallization, is a method for inducing the saturation of solutes in an aqueous solution using a water-miscible solvent to precipitate inorganic salts [264]. SDFC can be used to fractionate important resources, including nickel, cobalt, lithium, and rare earth elements, from industrial wastewater and solution mine leachate [265]. Thus, SDWE and SDFC can extract valuable minerals from contaminated wastewater without the need for water evaporation. These methods also protect existing freshwater resources by reducing the environmental impact of wastewater treatments and minimizing wastewater runoff.

DME has a high relative volume, which increases the number of separation steps in the solvent regeneration system and reduces residual solvent loss [266]. The most promising organic solvent candidates are organic compounds that form asymmetric hydrogen bonds with water, such as DME and trimethylamine. At 25 °C, these solvents exhibit volatilities that are an order of magnitude higher than that of water [262]. DME is a low-polarity organic solvent partially miscible with water. The low polarity of DME, with a dielectric constant less than 5.0, minimizes the solubility of electrolytes such as sodium chloride in the organic phase, enabling almost complete salt removal [108,263].

Moreover, the high volatility of DME allows for the rapid separation of water from the water–DME mixture after absorption [266]; the low boiling point of DME (−24.8 °C) minimizes the loss of foreign solvents in concentrated brine and demineralized water; DME is a good choice for H-donor and H-acceptor solvents compared to other solvents and has the advantage of being applicable to both solvent-driven fractional crystallization and fractional crystallization methods [262].

Stetson et al. separated rare-earth and transition-metal salts from industrially generated magnet waste via fractional distillation crystallization using DME [267]. Lanthanides and transition metals were selectively precipitated from aqueous solutions of metal salts by feeding DME gas at a high pressure and allowing them to dissolve. This method allows for the nontoxic separation of valuable elements from mixed salt solutions. The separation of metals is facilitated by the differential response of the solubility of transition metal and lanthanide sulfates to changes in temperature. Moreover, in the temperature range of 20–50 °C, the solubility of transition metal sulfates in water increases and that of lanthanide sulfates decreases.

## 8. Conclusions

This review focused on the use of liquefied DME as an eco-friendly solvent in various innovative extraction techniques. The low boiling point and solvent safety of DME enable the efficient extraction of diverse bioactive compounds from aqueous samples without prior drying. We also explored the superiority of liquefied DME extraction over conventional methods, explained its extraction mechanism, and highlighted its safety. This review discussed the potential of liquefied DME for various extraction processes and anticipated its future applications. Moreover, we discussed the estimation and trend analysis of the HSPs. This review highlighted the potential applications of DME in the extraction of functional compounds from various sources and offered a safer option than traditional toxic solvents for industries involved in natural product extraction.

## Figures and Tables

**Table 2:** Extraction system using commercially available liquefied DME.

Product Name	Specification	Solvents and Applications	Reference
Dexso Butanex 345/600 mm Extractor	A 125 or 50 cm extraction tube, for up to 40 g or 100 g (1.4 or 3.6 ounces) Centerpiece with magnetic tripod, easy assembly Magnetic feet, safe stand O-ring seal, safe extraction Emptying unit, easy emptying Reusable stainless-steel screen, low maintenance Temperature: room temperature	Supports DME and butane. Ideal for small amounts of plant material and trimming.	[141]
Pilot Extraction Plants	Volume extractor: 3 L Volume extract/solvent tank: 2 × 20 L Design temperature: −10/+50 °C	Supports DME, propane, and butane. Solvents can be reused/recycled.	[142]
Subcritical extraction equipment	Extraction tank volume: 2.5 L Production capacity: 20 L Temperature: <40 °C Using pressure: 0.8 MPa	Supports DME, butane, hexane, and ethanol. Ideal for plant oil extraction.	[143]
Mini solvent extraction unit for lab	Volume of extraction pot: 5–200 L Separation tank: 5–200 L Temperature: room temperature Buffer tank: 5–200 L Condenser: 1–10 m^2^ Solvent pot 19–159 L Gauge tank: 1–145 L Heater: 6 kw	Supports DME. Apply to precious vegetable oils, essential oils, animal oils, microalgae oils, natural dyes, vegetable proteins, and general-purpose spices. Solvents can be reused/recycled.	[144]

**Table 5:** Calculation of the HSP sphere model and RED for liquefied DME based on the HSPs of various solutes.

No.	Compounds	δd	δp	δh	RED	Reference
		(MPa^1/2^)	(MPa^1/2^)	(MPa^1/2^)		
Soluble						
1	Natural rubber	16.4	3.1	4.1	0.87	[218]
2	Nitrile rubber	20.4	12.4	4.1	0.88	[218]
3	Styrene-butadiene rubber	18	2.9	2.3	0.86	[219]
4	Ethylene-propylene rubber	17.2	2	2.6	0.94	[220]
5	Hydrogenated nitrile rubber	18.4	6	4.5	0.53	[221]
6	Fluoro rubber	16.1	9.3	6.6	0.78	[222]
7	Resveratrol	20.9	6.7	13.1	0.55	[204]
8	Phenanthrene	20.8	2.6	5.4	0.66	[223]
9	Pyrene	22.5	1.6	4	1.02	[223]
10	Lecithin	16.1	6.4	9.1	0.66	[224]
11	Camphor	17.3	10	4.9	0.73	SMILES
12	Ferulic acid	19.3	8.4	15.8	0.74	[225]
13	Caffeine	19.5	10.1	13	0.58	[226]
14	Curcumin	18.8	7.7	11.1	0.27	[227]
15	γ-oryzanol	18.6	6.5	3.3	0.64	[228]
16	Phytosterol	17.1	1.9	3	0.92	SMILES
17	Policosanol	15.9	1.7	4.4	1.00	[229]
18	Triterpenoid	18	9.2	12.8	0.55	SMILES
19	*trans*-Resveratrol	20.6	7.3	15.9	0.78	[230]
20	β-carotene	17.1	2.4	5.5	0.73	[231]
21	Policosanol	16.1	2.4	5	0.90	[229]
22	Oleic acid	16	2.8	6.2	0.84	[232]
23	Linoleic acid	18.1	2.9	7.2	0.48	[233]
24	Lutein	15.2	1.8	8.5	0.98	[234]
25	Xanthone	20.6	8.4	5.2	0.57	SMILES
26	Fucoxanthin	18.2	4.1	5.1	0.54	[235]
27	Astaxanthin	22.2	4.6	8.9	0.66	[236]
28	Capsaicin	18.3	15.4	8.9	0.99	SMILES
29	Butyl rubber	17.3	1.4	2.6	0.96	[218]
Insoluble						
30	Chloroprene rubber	17.2	2.4	1.2	1.04	SMILES
31	Polytetrafluoroethylene	16.2	1.8	3.4	1.01	[237]
32	Low density polyethylene	16.2	2.1	2.4	1.06	[238]
33	Polyvinyl alcohol	17	9	18	1.09	Solubility experiments
34	Polyvinylpyrrolidone	18.1	10	18	1.04	Solubility experiments
35	Chitosan	22.8	17.1	26.6	2.31	Solubility experiments
36	Chitin	23.3	15	22.5	1.90	[239]
37	Polyacrylamide	19.5	19.7	16.4	1.62	Solubility experiments
38	Polyglutamic acid sodium salt	19.3	12.1	16.5	0.99	Solubility experiments

## Data Availability

Data are contained within the article.
